# Synthesis of dumbbell-like ZnO microcrystals via a simple solution route

**DOI:** 10.1186/1556-276X-7-507

**Published:** 2012-09-11

**Authors:** Zhenqing Hou, Yixiao Wang, Lihua Shen, Hao Guo, Gongxin Wang, Yang Li, Shuifan Zhou, Qiqing Zhang, Qian Jiang

**Affiliations:** 1 Research Center of Biomedical Engineering, Department of Biomaterials, College of Materials, Xiamen University, The Key Laboratory of Biomedical Engineering of Fujian Province, Research Center of Biomedical Engineering and Technology of Xiamen City, Xiamen, Fujian 361005, PR China; 2 Air Force General Hospital, 343 PLA, Beijing 100142, China; 3 Institute of Biomedical Engineering, Chinese Academy of Medical Science, Peking Union Medical College, The Key Laboratory of Biomaterial Material of Tianjin, Tianjin 300192, PR China

**Keywords:** ZnO, Dumbbell-like, Micro-rods, Solution route

## Abstract

Uniform dumbbell-like ZnO microcrystals had been successfully fabricated on a large scale via a facile solution technique under mild conditions. Obtained ZnO, with length of 1.2 to 1.6 μm and diameters of 350 to 600 nm, exhibited well-defined dumbbell-like morphology and hexagonal wurtzite structure and grew along the [001] direction. Effects of the reactant concentration on the sizes and morphologies of the ZnO products had been investigated, indicating that the reactant concentration played a crucial role in determining final sizes and shapes of the samples. In addition, the growth process of the dumbbell-like ZnO microcrystals was studied, and a possible formation mechanism was proposed. Furthermore, the optical properties of ZnO samples obtained at various reaction times were also investigated by photoluminescence (PL) spectroscopy. The PL spectra of the as-prepared dumbbell-like ZnO microcrystals showed a strong UV emission peak.

## Background

As a wide bandgap (3.37 eV) and large exciton binding energy (60 meV) at room temperature, ZnO is recognized as one of the most important photonic materials for applications in electronic devices such as piezoelectric transducers [1], blue light-emitting diodes [2-4], solar cells [5-7], and gas sensors [8,9]. Substantial efforts have been employed in controlling the morphology, size, and dimension of ZnO crystals because these parameters represent key elements that largely determine the electronic and optical performances of ZnO. In particular, the synthesis of one-dimensional ZnO nano- or microstructures in shapes including rods, tubes, needles, dumbbells, and wires has attracted immense attentions from the research communities due to their potential applications in the optoelectronic devices and functional materials. Different techniques, such as the reaction of zinc salts with base [10-12], the wet chemical bath deposition [13-15], solvothermal [16], chemical vapor deposition [17], template methods [18-21], hydrothermal [22-26], etc., have been exploited to prepare one-dimensional ZnO nano- or microstructure materials with various morphologies. Among these methods, vapor-phase processes are expensive and energy-consuming, which are not suitable for large scale production. The solution-based methods, however, demonstrate obvious advantages of low-cost, low-temperature operation to prepare large-scale and well-crystallized ZnO materials.

Recently, Riley and co-workers had synthesized ZnO one-dimensional nanostructures on Si wafers coated with a thin film of ZnO via a hydrothermal method [27]. However, the synthesis process was comparatively complicated, which was not appropriate for large-scale production. Hu and co-workers had reported the synthesis of ZnO nanowires and nanobelts on a large scale using ZnCl_2_ as zinc source, Na_2_CO_3_ as mineralizer, and sodium dodecyl sulfonate as morphology controller agent via a low-temperature one-pot hydrothermal technique [28]. However, the reaction time was too long. Zhang et al. had prepared dumbbell-like ZnO microcrystals of hexagonal phase using poly (vinyl alcohol) as the capping molecules at 200°C for 12 h [29]. However, the morphologies of the ZnO crystals were controlled by additives and still required a high temperature. Although progresses have been made in the synthesis of one-dimensional ZnO, a simple and fast approach had remained a great challenge.

In this paper, a conventional technique was utilized to simply prepare one-dimensional ZnO. The highly uniformed dumbbell-like ZnO could be easily fabricated via a simple low-temperature solution route. This synthetic approach also allowed further reducing the growth temperature to 95°C and shortening the reaction time to 3 h, leading to the development of an effective and low-cost fabrication process for high-quality ZnO. Moreover, we reported the low-temperature synthesis of dumbbell-like ZnO, which synthesized at ambient pressure without any substrates or additives. The morphology, structure, and properties of the obtained ZnO were examined; the effects of the reactant concentration and reaction time on the size and shapes of the ZnO products were analyzed, and the possible growth mechanism of the ZnO microstructure was discussed. Furthermore, the room temperature UV–vis absorption and photoluminescence (PL) spectrum of the obtained products were also investigated.

## Methods

All of the reactants and solvents were of analytical grade and used as received without any further purification. In a typical synthesis process, 50 mL of aqueous solution of Zinc nitrate hexahydrate (Zn(NO_3_)_2_·6H_2_O) and 50 mL of hexamethylenetetramine ((CH_2_)_6_ N_4_, HMT) aqueous solution of equal concentration (0.25 M) were mixed together. Then, the mixture was heated at 95°C for 3 h under mild magnetic stirring with refluxing, followed by cooling to room temperature naturally. Subsequently, the resulting white products were washed with deionized water, and dried at 60°C in the air for further characterization.

Transmission electron microscope (TEM) images and selected area electron diffraction (SAED) patterns were obtained on a JEOL JEM-2100 (Tokyo, Japan) operated at an accelerating voltage of 200 kV. Samples for TEM and high-resolution transmission electron microscope (HR-TEM) analyses were prepared by spreading a drop of as-prepared magnetite nanoparticle dilute dispersion on copper grids coated with a carbon film followed by evaporation under ambient conditions. The scanning electron microscopy (SEM) images were obtained on a LEO 1530 microscope (LEO, Germany). Atomic force microscope (AFM) characterization was carried out using a Scan Asyst-Air (Bruker Multimode 8, Bruker ASX Inc., Madison, WI, USA). Measurements were carried out in air using non-contact AFM mode. The X-ray diffraction (XRD) patterns were collected between 20° and 80° (2*θ*) on an X-ray diffraction system (X’Pert PRO, PANalytical Co., Almelo, The Netherlands) with a graphite monochromator and Cu Kα radiation (*λ* = 0.15406 nm). X-ray photoelectron spectrum (XPS) was obtained using a PHI Quantum-2000 electron spectrometer (Physical Electronics, Inc., Chanhassen, MN, USA) with 150-W monochromatized Al Kα radiation (1,486.6 eV). The PL emission spectra were recorded using an Edinburgh FLS 920 fluoresence spectrophotometer (Xe 900 lamp) (Edinburgh Photonics, Livingston, UK) at room temperature. The excitation wavenumber was 290 nm. The optical absorption was measured with an ultraviolet–visible (UV–vis) spectrophotometer (UV, BECKMAN COULTER DU 800, Conquer Scientific, San Diego, CA, USA) at room temperature.

## Results and discussion

### Structure and morphology

**Figure 1:**
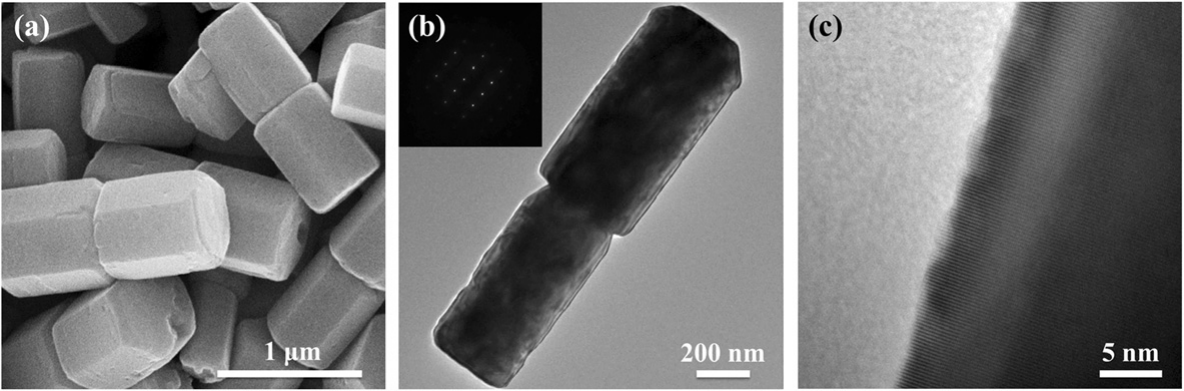
**SEM and TEM images of the dumbbell-like ZnO sample.** (**a**) SEM image, (**b**) TEM image and SAED pattern (inset), and (**c**) HR-TEM image of the sample.

**Figure 2:**
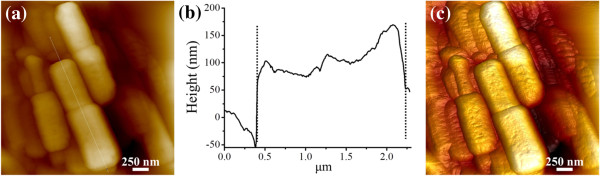
**AFM height image, height profile, and 3D image reconstruction.** (**a**) AFM height image of ZnO sample on mica. (**b**) Height profile along the white line in Figure 2a. (**c**) 3D image reconstruction of ZnO sample.

The morphology of the as-prepared ZnO was characterized by SEM and TEM (Figure 1). As shown in Figure 1a, the dumbbell structured ZnO consisted of two hexagonal ZnO microcrystals. The dumbbell-like ZnO had diameters ranging between 350 and 600 nm and lengths of 1.2 to 1.6 μm. Figure 1b showed the TEM image of the dumbbell-like ZnO microcrystals. The inset area electron diffraction (SAED) pattern performed on the individual dumbbell-like ZnO microcrystal indicated that the dumbbell-like ZnO was a single crystal and could be indexed as the hexagonal ZnO phase. The HR-TEM image recorded at the edge of the dumbbell-like ZnO was shown in Figure 1c, indicating well-defined lattice fringes with interplanar spacing of 0.260 nm for (0002) plane of hexagonal structured ZnO. The surface morphology of the as-prepared dumbbell-like ZnO microcrystals was further shown in Figure 2. Figure 2a,c showed the 2D and 3D AFM images of ZnO samples. The ZnO sample appeared as hexagonal dumbbell-structured microcrystals, which were quite in agreement with the results obtained from TEM images. In addition, Figure 2c provided a height profile along the thin white line in Figure 2a. It was obvious that the height was fluctuant, indicating that the surface of the as-prepared dumbbell-like ZnO microcrystals was not totally flat.

### Effect of the reaction conditions on the ZnO microstructures

#### Effect of the reaction concentration

**Figure 3:**
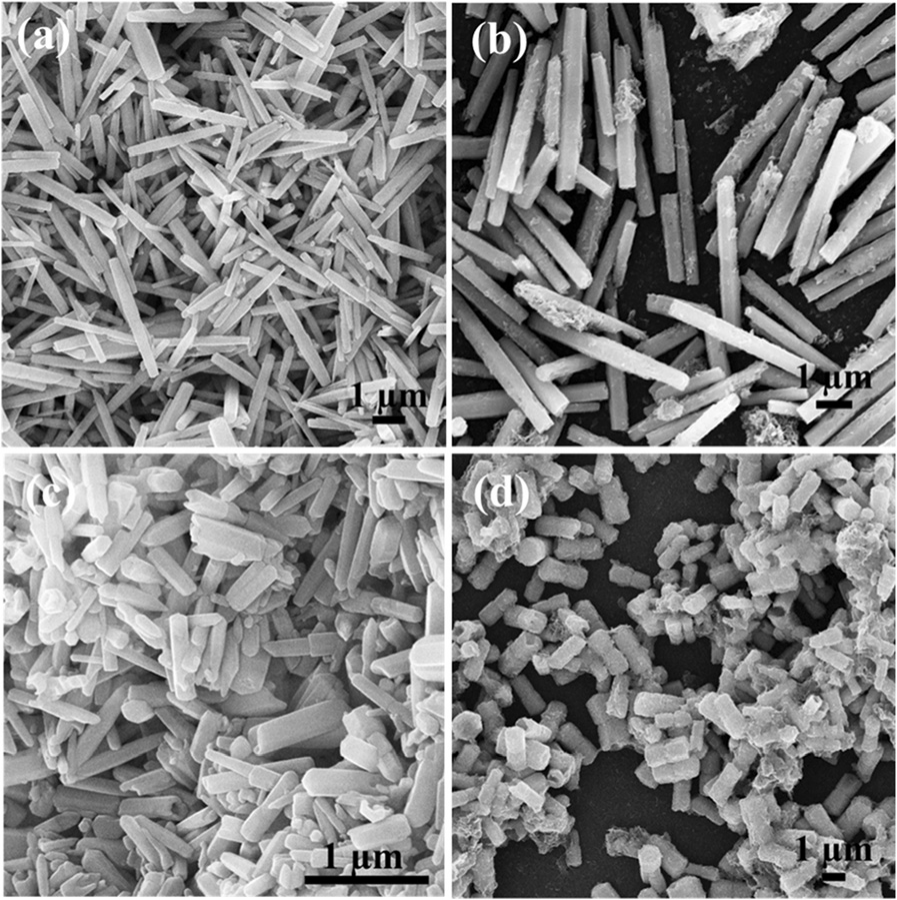
**SEM images of the synthesized ZnO samples.** With different reactant concentrations: (**a**) 0.01, (**b**) 0.03, (**c**) 0.1, and (**d**) 0.5 M. The reaction time was 180 min.

To investigate the effect of the reactant concentration, we changed the concentration of the Zn(NO_3_)_2_·6H_2_O and HMT while the other parameters were kept constant. The morphologies of the synthesized ZnO samples at different concentration were shown in Figure 3. When the reaction was run at a low concentration (0.01 M), the obtained ZnO sample exhibited the typical micro-rod structure with an aspect ratio of 18 to 30 and a diameter of 200 to 400 nm. When the concentration was increased to 0.03 M, ZnO micro-rods were obtained with a smaller aspect ratio of 6 to 12 and a larger diameter of 400 to 700 nm, indicating that ZnO micro-rods became shorter and wilder as the concentration increased. Especially when the concentration was increased to 0.1 M, the obtained ZnO sample showed the dumbbell-like structure, but the dumbbell-like structures were asymmetrical. Once the concentration was increased to 0.25 M, monodisperse, dumbbell-like ZnO microcrystal was successfully obtained (Figure 1). When the concentration further increased to 0.5 M, the typical hexagonal dumbbell-like crystals with an aspect ratio of 4 to 8 and a diameter of 500 to 900 nm were obtained.

#### Effect of the reaction time

**Figure 4:**
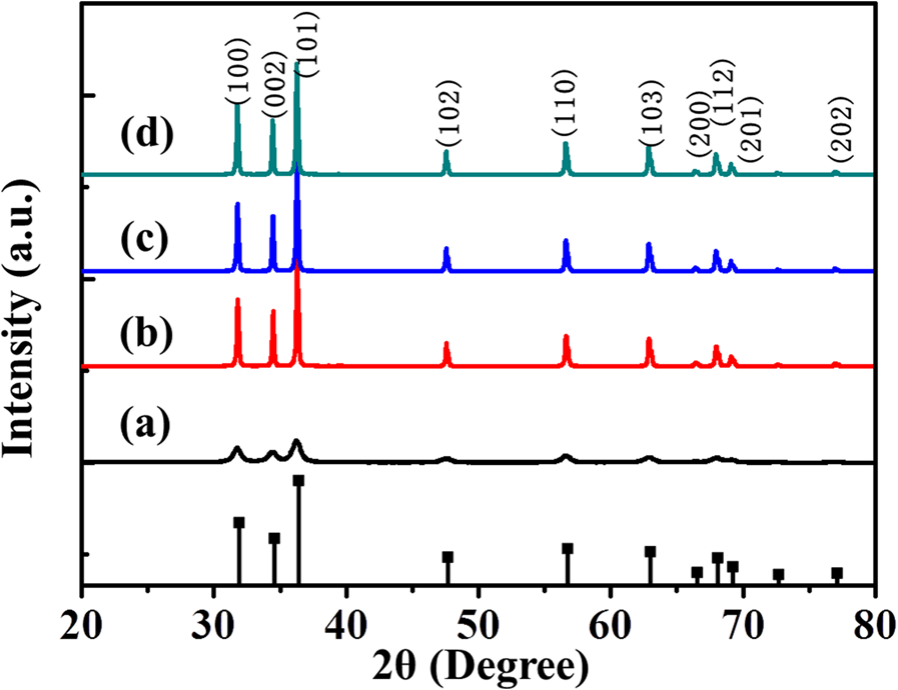
**XRD patterns of the ZnO samples.** Obtained at different reaction times: (**a**) 10, (**b**) 60, (**c**) 120, and (**d**) 180 min. The reactant concentration was 0.25 M.

In order to investigate the formation process of the dumbbell-like ZnO microcrystal, the ZnO samples were collected after 10, 60, 120, and 180 min, respectively. Figure 4 showed the XRD patterns of the samples synthesized at various times, and the diffraction peaks could be perfectly indexed to a hexagonal (wurtzite) structure of ZnO (JCPDS 01-089-7102). No characteristic peak of impurities was detected in the pattern. Moreover, the peak intensities of the as-obtained samples steadily became stronger with increasing reaction time, indicating that the longer time reaction led to higher crystal quality.

**Figure 5:**
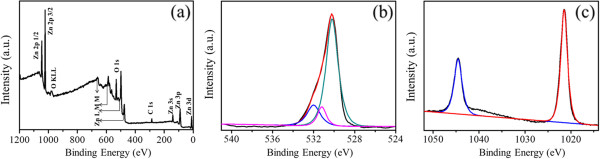
**XPS spectra of the ZnO sample.** (**a**) Survey spectrum of the sample, (**b**) O 1 *s* spectrum, and (**c**) Zn 2*p* spectrum.

The surface element composition of the as-obtained sample was further studied by XPS analysis. Figure 5 showed the survey of the sample. No peaks of other elements except Zn, C, and O were detected. The presence of C could be mainly attributed to the atmospheric contamination due to the exposure of the sample to air. The binding energies in all the XPS spectra were calibrated using that of C 1 *s*. The high-resolution spectra of O and Zn regions were also displayed in Figure 2b,c, respectively. The O 1 *s* peak was centered at 530.8 eV. Also, the binding energies of Zn 2*p*_3/2_ and Zn 2*p*_1/2_ were 1,021.7 and 1,044.8 eV, respectively. All these binding energies were very close to the standard values of the bulk ZnO [30]. Both XRD and XPS analyses indicated that the as-prepared products were pure ZnO. 

**Figure 6:**
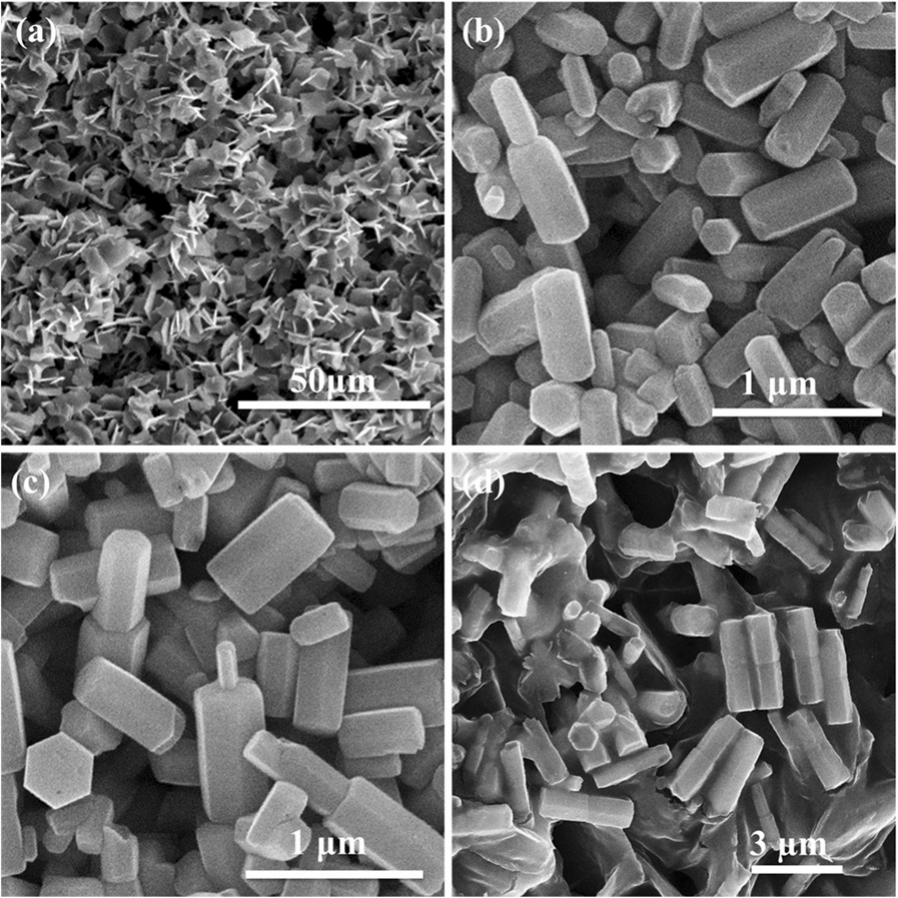
**SEM images of the synthesized ZnO samples.** With different reaction times: (**a**) 10, (**b**) 60, (**c**) 120, and (**d**) 180 min. The reaction concentration was 0.25 M.

The microstructures of the samples collected at different times were then observed by SEM (Figure 6). The samples aged at 95°C for only 10 min revealed that lots of lamella aggregates constructed with small lamellas were formed, and little ZnO micro-rods could be observed. As the reaction time was prolonged to 60 min, the lamella aggregation decreased, while a number of ZnO micro-rods began to form. However, these ZnO microcrystals were rod-like structures rather than dumbbell-like ones. This suggested that ZnO lamellas were only an intermediate and would gradually form ZnO micro-rods as the reaction time increased. Further prolonging the reaction time to 120 min, amounts of the dumbbell-like microcrystals were obtained. The microcrystals were asymmetrical because one subunit was larger than the other. When the reaction proceeded to 180 min, the microcrystals grow larger and longer. Individual dumbbell-like ZnO microcrystals were obtained.

#### Growth mechanism

To understand the growth behavior of the dumbbell-like ZnO microcrystal, it was necessary to investigate its growth mechanism. The reaction for the growth of ZnO crystal may be simply expressed as follows:

(1) $${\left({\text{CH}}_{2}\right)}_{6}{\text{N}}_{4}+4{\text{H}}_{2}\text{O}↔{\left({\text{CH}}_{2}\right)}_{6}{\text{N}}_{4}-4{\text{H}}^{+}+4{\text{OH}}^{-}$$

(2) $${\text{Zn}}^{2+}+4{\text{OH}}^{-}↔{{\text{ZnO}}_{2}}^{2-}+2{\text{H}}_{2}\text{O}$$

(3) $${{\text{ZnO}}_{2}}^{2-}+{\text{H}}_{2}\text{O}↔\text{ZnO}+2{\text{OH}}^{-}$$

In the synthesis systems, HMT acted as alkaline source to release OH^−^, which subsequently reacted with Zn^2+^ to form ZnO_2_^2−^. At the last period of the reaction, ZnO was largely formed via homogeneous precipitation under mild conditions.

**Figure 7:**
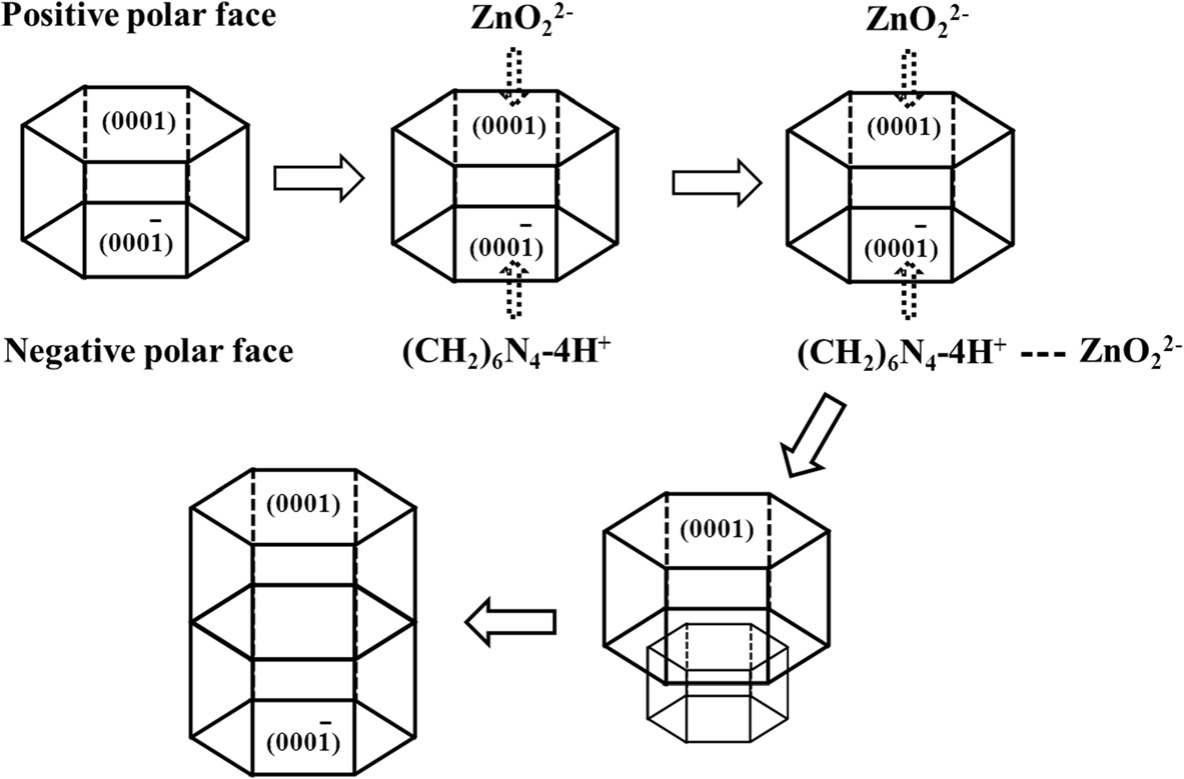
Illustration of the growth mechanism of the dumbbell-like ZnO.

Notably, the hexagonal ZnO crystal had both polar and nonpolar faces. Polar faces with surface dipoles were thermodynamically less stable than nonpolar faces and, therefore, needed to rearrange to reduce their surface energy. As illustrated in Figure 7, in the initial phase of reaction, ZnO_2_^2−^ ions were likely adsorbed on the positive polar face of the (0001) surface, making the growth along the [001] direction more faster, and thus, solid ZnO seed crystal appeared. In addition, HMT would hydrolyze in the aqueous solution, resulting in the formation of (CH_2_)_6_ N_4_-4 H^+^ complex, which would adsorb on the negative polar face of the surface due to the coulomb interaction. While ZnO_2_^2−^ ions were adsorbed on the (CH_2_)_6_ N_4_-4 H^+^ complex, a new seed crystal was formed. As the reaction time was prolonged, the seed crystals grew larger and longer, and finally, typical dumbbell-like ZnO microcrystals were formed. Many studies with similar morphologies had proposed the different growth mechanism. When an inversion boundary was formed in ZnO, it predominantly showed the head-to-head configuration of the c polar axis, i.e., the oxygen-terminated planes pointing towards each other with the (0001) plane at the interface, leading to a dumbbell-like shape of grown rods [31]. In another experiment [32], most of Zn^2+^ ions were precipitated into Zn(OH)_2_. Under hydrothermal conditions, the Zn(OH)_2_ precipitates were transformed into growth units of [Zn(OH)_4_^2-^. In the presence of CTAB, the growth units could be bonded by the CTAB at the composition plane (0001) to form the nucleus of hexagonal cylinder-like twinning crystal. The growth of the individual crystallite in the twin crystal took place along the polar c-axis by means of the incorporation of the growth units on the growth interface (0001), and thus, hexagonal cylinder-like ZnO twinning crystals were formed ultimately. 

**Figure 8:**
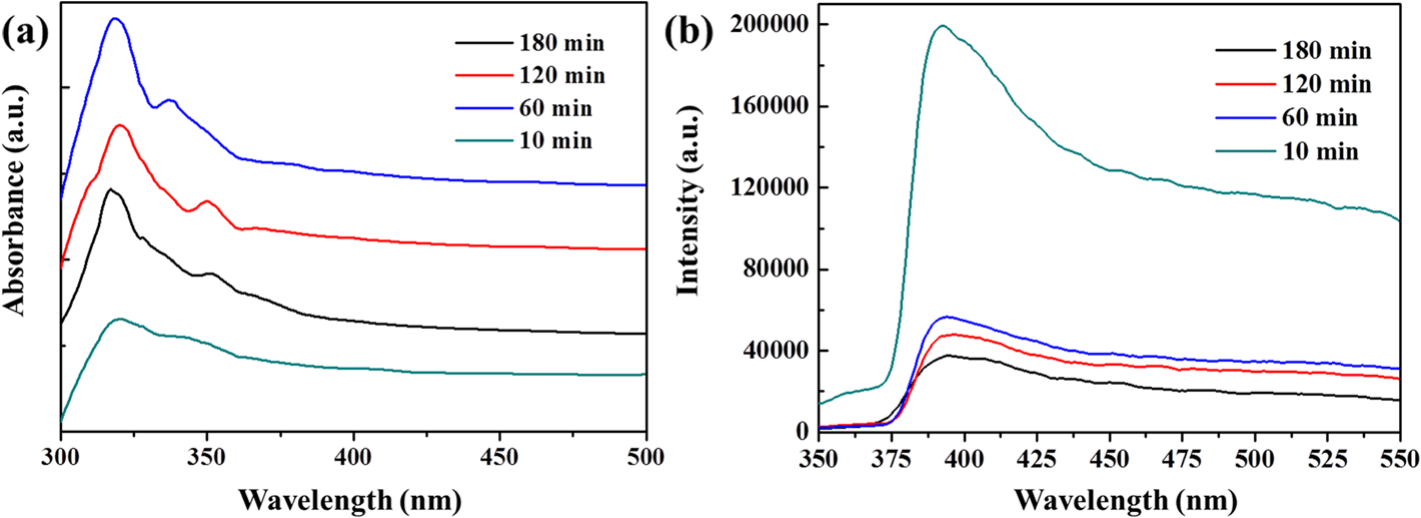
**UV–vis absorption spectra and room temperature PL spectra.** UV–vis absorption spectra of the products synthesized at 95°C for different reaction times (**a**) and room temperature PL spectra of the ZnO samples prepared at different reaction times (**b**).

Figure 8a showed the room temperature UV–vis absorption spectra of the samples collected at various reaction times. It could be observed that each UV–vis absorption spectrum consisted of a strong and broad absorption band from ultraviolet to visible region, and the maximum absorption peak appeared at 320 nm. In addition, it could be found that the absorption of the samples were dependent on the reaction time. The longer reaction time led to the stronger absorbance, which could be attributed to the change of crystal quality and morphology as the reaction time prolongs. Considering the excellent optional properties of the as-prepared samples, they may have a wide potential application in the photocatalytic field.

The PL from ZnO were always composed of two emission bands at room temperature: a near-band-edge (UV) emission and a broad, deep-level (visible) emission. The visible emission was usually ascribed to various intrinsic defects produced during the synthesis of ZnO, such as zinc vacancy and oxygen vacancy [33,34]. Figure 8b showed the room temperature PL spectra of the ZnO samples collected at different times. The PL spectra of the samples collected at reaction times of 10, 60, 120, and 180 min revealed similar features. There appeared a strong broad UV emission at approximately 392 nm, which was the band-edge emission resulting from the recombination of free excitons. No other peaks such as green emission (approximately 520 nm) were found. It should be mentioned that the green emission resulted from the radiative recombination of a photogenerated hole with electron occupying the oxygen vacancy [35]. It was commonly accepted that high-quality ZnO crystals would only emit UV light. Therefore, it was reasonably believed that the as-prepared dumbbell-like ZnO microcrystals were of good quality and may have a wide application in the photocatalytic field.

## Conclusion

In summary, single crystalline ZnO with hexagonal dumbbell-like microstructure had been successfully synthesized via a facile solution method under mild conditions without any additives, templates, or substrates. The effects of the reactant concentration on the size and shapes of the ZnO samples were studied, and the results indicated that the ZnO preferential growth difference between [001] and other directions would decrease with increasing the reaction concentration. The growth mechanism of the dumbbell-like ZnO microcrystals was discussed in the point of nucleation and morphology view. Moreover, the dumbbell-like ZnO microcrystals had a very strong UV emission at approximately 392 nm, which might be very interesting for further application to microscale optoelectronic devices due to their excellent UV emission properties.

## Competing interests

The authors declare that they have no competing interests.

## Authors’ contributions

ZH, YW, and QZ conceived the study and drafted the manuscript. LS, HG, and GW carried out the part of the experiments. YL and SZ participated in the sequence alignment. QJ participated in the design of the study and drafted the part of the manuscript. All authors read and approved the final manuscript.

## Authors’ information

ZH and QJ are Ph.Ds. and associate professors. YW, LS, HG, GW, YL, and SZ are M.D. students in the Research Center of Biomedical Engineering. QZ is a Ph.D. and a professor.

## References

[B1] Tu Z Hu X Elasticity and piezoelectricity of zinc oxide crystals, single layers, and possible single-walled nanotubes Phys Rev B 2006 74 3 035434

[B2] Huang MH Mao S Feick H Yan H Wu Y Kind H Weber E Russo R Yang P Room-temperature ultraviolet nanowire nanolasers Science 2001 292 5523 1897 1899 10.1126/science.1060367 11397941

[B3] Bao J Zimmler MA Capasso F Wang X Ren Z Broadband ZnO single-nanowire light-emitting diode Nano Lett 2006 6 8 1719 1722 10.1021/nl061080t 16895362

[B4] Wang M Fei GT De Zhang L Porous-ZnO-nanobelt film as recyclable photocatalysts with enhanced photocatalytic activity Nanoscale Res Lett 2010 5 11 1800 1803 10.1007/s11671-010-9715-x 21124630 PMC2964478

[B5] Law M Greene LE Johnson JC Saykally R Yang P Nanowire dye-sensitized solar cells Nat Mater 2005 4 6 455 459 10.1038/nmat1387 15895100

[B6] Keis K Magnusson E Lindström H Lindquist SE Hagfeldt A A 5% efficient photoelectrochemical solar cell based on nanostructured ZnO electrodes Sol Energy Mater Sol Cells 2002 73 1 51 58 10.1016/S0927-0248(01)00110-6

[B7] Luan C Vaneski A Susha AS Xu X Wang HE Chen X Xu J Zhang W Lee CS Rogach AL Facile solution growth of vertically aligned ZnO nanorods sensitized with aqueous CdS and CdSe quantum dots for photovoltaic applications Nanoscale Res Lett 2011 6 1 1 8 10.1186/1556-276X-6-340 PMC3211429 21711865

[B8] Wang XL Chao H Li H Hong XL Ji LN Li XY Synthesis, crystal structure and DNA cleavage activities of copper (II) complexes with asymmetric tridentate ligands J Inorg Biochem 2004 98 3 423 429 10.1016/j.jinorgbio.2003.12.006 14987842

[B9] Wang CY Wu HD Lee LN Chang HT Hsu YL Yu CJ Yang PC Hsueh PR Pasteurization is effective against multidrug-resistant bacteria Am J Infect Control 2006 34 5 320 322 10.1016/j.ajic.2005.11.006 16765213

[B10] Calestani D Zha M Zanotti L Villani M Zappettini A Low temperature thermal evaporation growth of aligned ZnO nanorods on ZnO film: a growth mechanism promoted by Zn nanoclusters on polar surfaces Cryst Eng Comm 2011 13 5 1707 1712 10.1039/c0ce00670j

[B11] Lin Y Jiang Q Effect of substrates and anions of zinc salts on the morphology of ZnO nanostructures Appl Surf Sci 2011 20 257 8728 8731

[B12] Akgun MC Afal A Unalan HE Hydrothermal zinc oxide nanowire growth with different zinc salts J Mater Res 2012 1 1 1 7

[B13] Li Q Bian J Sun J Wang J Luo Y Sun K Yu D Controllable growth of well-aligned ZnO nanorod arrays by low-temperature wet chemical bath deposition method Appl Surf Sci 2010 256 6 1698 1702 10.1016/j.apsusc.2009.09.097

[B14] Liu X Afzaal M Badcock T Dawson P O’Brien P Conducting ZnO thin films with an unusual morphology: Large flat microcrystals with (0 0 0 1) facets perpendicular to the plane by chemical bath deposition Mater Chem Phys 2011 1-2 127 174 178

[B15] Kathalingam A Ambika N Kim M Elanchezhiyan J Chae Y Rhee J Chemical bath deposition and characterization of nanocrystalline ZnO thin films Mater Sci Pol 2010 28 2 513 522

[B16] Wu J Shen X Jiang L Wang K Chen K Solvothermal synthesis and characterization of sandwich-like graphene/ZnO nanocomposites Appl Surf Sci 2010 256 9 2826 2830 10.1016/j.apsusc.2009.11.034

[B17] Bekermann D Gasparotto A Barreca D Bovo L Devi A Fischer RA Lebedev OI Maccato C Tondello E Van Tendeloo G Highly oriented ZnO nanorod arrays by a novel plasma chemical vapor deposition process Crystal growth & design 2010 10 4 2011 2018 10.1021/cg1002012

[B18] Wang X Zhang Q Wan Q Dai G Zhou C Zou B Controllable ZnO Architectures by Ethanolamine-Assisted Hydrothermal Reaction for Enhanced Photocatalytic Activity J Phys Chem C 2011 115 6 2769 2775 10.1021/jp1096822

[B19] Erdlyi R Nagata T Rogers D Teherani FH Horvth ZE L Badi Z Baji Z Wakayama Y Volk J Investigations into the impact of the template layer on ZnO nanowire arrays made using low temperature wet chemical growth Crystal growth & design 2011 11 6 2515 2519 10.1021/cg2002755

[B20] Umar A Ribeiro C Al-Hajry A Masuda Y Hahn YB Growth of highly c-axis-oriented ZnO nanorods on ZnO/Glass substrate: growth mechanism, structural, and optical properties J Phys Chem C 2009 113 33 14715 14720 10.1021/jp9045098

[B21] Tian Y Li J Xiong H Dai J Controlled synthesis of ZnO hollow microspheres via precursor-template method and its gas sensing property Appl Surf Sci 2011 22 258 8431 8438

[B22] Hu Y Qian H Liu Y Du G Zhang F Wang L Hu X A microwave-assisted rapid route to synthesize ZnO/ZnS core–shell nanostructures via controllable surface sulfidation of ZnO nanorods Cryst Eng Comm 2011 13 10 3438 3443 10.1039/c1ce05111c

[B23] Zhang R Yang X Zhang D Qin J Lu C Ding H Yan X Tang H Wang M Zhang Q Facile morphology‐controlled hydrothermal synthesis of flower‐like self‐organized ZnO architectures Cryst Res Technol 2011 11 46 1189 1194

[B24] Gao P Wang L Wang Y Chen Y Wang X Zhang G One‐pot hydrothermal synthesis of heterostructured ZnO/ZnS nanorod arrays with high ethanol‐sensing properties Chemistry-a European Journal 2012 18 15 4681 4686 10.1002/chem.201102927 22407815

[B25] Wu Q Chen X Zhang P Han Y Yan Y Li S Amino acid-assisted synthesis of ZnO hierarchical architectures and their novel photocatalytic activities Crystal Growth and Design 2008 8 8 3010 3018 10.1021/cg800126r

[B26] Liu C Li H Jie W Zhang X Yu D Preparation of ZnO cluster and rod-like whiskers through hydrothermal methods Mater Lett 2006 60 11 1394 1398 10.1016/j.matlet.2005.11.035

[B27] Ashfold MNR Doherty RP Ndifor-Angwafor NG Riley DJ Sun Y The kinetics of the hydrothermal growth of ZnO nanostructures Thin Solid Films 2007 515 24 8679 8683 10.1016/j.tsf.2007.03.122

[B28] Hu H Huang X Deng C Chen X Qian Y Hydrothermal synthesis of ZnO nanowires and nanobelts on a large scale Mater Chem Phys 2007 106 1 58 62 10.1016/j.matchemphys.2007.05.016

[B29] Zhang H Yang D Li D Ma X Li S Que D Controllable growth of ZnO microcrystals by a capping-molecule-assisted hydrothermal process Crystal growth & design 2005 5 2 547 550 10.1021/cg049727f

[B30] Peng W Qu S Cong G Wang Z Synthesis and structures of morphology-controlled ZnO nano-and microcrystals Crystal growth & design 2006 6 6 1518 1522 10.1021/cg0505261

[B31] Wang B Shi E Zhong W Twinning morphologies and mechanisms of ZnO crystallites under hydrothermal conditions Cryst Res Technol 1998 33 6 937 941 10.1002/(SICI)1521-4079(1998)33:6<937::AID-CRAT937>3.0.CO;2-8

[B32] Liu Y Lv H Li S Xi G Xing X Synthesis and characterization of ZnO microstructures via a cationic surfactant-assisted hydrothermal microemulsion process Mater Charact 2011 5 62 509 516

[B33] Dev A Kar S Chakrabarti S Chaudhuri S Optical and field emission properties of ZnO nanorod arrays synthesized on zinc foils by the solvothermal route Nanotechnology 2006 17 1533 10.1088/0957-4484/17/5/061

[B34] Kar S Dev A Chaudhuri S Simple solvothermal route to synthesize ZnO nanosheets, nanonails, and well-aligned nanorod arrays J Phys Chem B 2006 110 36 17848 17853 10.1021/jp0629902 16956271

[B35] Vanheusden K Warren W Seager C Tallant D Voigt J Gnade B Mechanisms behind green photoluminescence in ZnO phosphor powders J Appl Phys 1996 79 10 7983 7990 10.1063/1.362349

